# Substantia nigra degeneration in spinocerebellar ataxia 2 and 7 using neuromelanin‐sensitive imaging

**DOI:** 10.1111/ene.70035

**Published:** 2025-01-05

**Authors:** Lydia Chougar, Giulia Coarelli, François‐Xavier Lejeune, Pia Ziegner, Rahul Gaurav, Emma Biondetti, Sabrina Sayah, Rania Hilab, Alain Dagher, Alexandra Durr, Stéphane Lehéricy

**Affiliations:** ^1^ Institut du Cerveau–Paris Brain Institute ICM, Sorbonne Université, Inserm 1127, CNRS 7225, Hôpital de la Pitié Salpêtrière Paris Paris France; ^2^ Department of Neuroradiology Hôpital Pitié‐Salpêtrière Paris France; ^3^ The Neuro (Montreal Neurological Institute‐MNI), McGill University Montreal Canada; ^4^ Sorbonne Université, Paris Brain Institute's Data Analysis Core Facility, Inserm, CNRS, APHP, Hôpital de la Pitié‐Salpêtrière Paris France; ^5^ Department of Neurosciences, Imaging, and Clinical Sciences University ‘G. D'Annunzio’ of Chieti‐Pescara Chieti Italy; ^6^ Institute for Advanced Biomedical Technologies, University ‘G. D'Annunzio’ of Chieti‐Pescara Chieti Italy

**Keywords:** spinocerebellar ataxia, parkinsonism, biomarker, MRI, neuromelanin, substantia nigra

## Abstract

**Objective:**

Spinocerebellar ataxias (SCA) are neurodegenerative diseases with widespread lesions across the central nervous system. Ataxia and spasticity are usually predominant, but patients may also present with parkinsonism. We aimed to characterize substantia nigra *pars compacta* (SNc) degeneration in SCA2 and 7 using neuromelanin‐sensitive imaging.

**Methods:**

Ataxic and preataxic expansion carriers with SCA2 (n=15) and SCA7 (n=15) and healthy controls (n=10) were prospectively recruited. Volume and signal‐to‐noise ratio (SNR) values of the SNc were extracted from neuromelanin‐sensitive images. ROC curves were used to determine the metrics that best differentiated SCA participants. Correlations between imaging measurements, clinical variables, and plasma neurofilaments light chain (NfL) levels were investigated.

**Results:**

SCA2 participants had lower SNR values in the SNc than controls (110.2 ± 1.3 versus 113.2 ± 1.4; *p* < 0.001) and those with SCA7 (112.5 ± 2.1; *p* < 0.01). SNR in SCA7 participants and controls did not differ. In ataxic patients, SNc volumes were lower in SCA2 (0.13 ± 0.04; *p* = 0.06) and SCA7 (0.10 ± 0.03, *p* = 0.02) patients compared to controls (0.17 ± 0.04). Signal decrease was detected at the preataxic stage in SCA2, but not in SCA7. SCA2 participants showed prominent involvement of the associative and limbic nigral territories. SNR discriminated ataxic and preataxic SCA2 participants from controls (AUC ≥0.94). SNc volume differentiated ataxic SCA7 participants from controls (AUC = 1), but not preataxic ones. In SCA7, correlations were observed between SNc volume and time to onset, CAG repeats, clinical severity scores, and NfL.

**Conclusions:**

Neuromelanin‐sensitive imaging provides biomarkers of nigral degeneration in SCAs, detectable from the preataxic stage in SCA2, which could potentially serve as outcome measures in clinical trials.

## INTRODUCTION

Polyglutamine spinocerebellar ataxias (SCAs) are autosomal dominant neurodegenerative diseases caused by CAG trinucleotide repeat expansions [1, 2, 3]. These subtypes are characterized by degeneration of the brainstem and cerebellum, which can be detected before the onset of ataxia [4, 5]. Imaging biomarkers are of particular interest in the preataxic phase, as clinical scales are not sufficiently sensitive at this stage. Neuromelanin‐sensitive imaging is a magnetic resonance imaging (MRI) technique that relies on the paramagnetic properties of neuromelanin in dopaminergic neurons [6], and enables the study of the substantia nigra *pars compacta* (SNc) neurodegeneration. This technique has been widely applied in the study of Parkinsonism [7, 8, 9, 10, 11]. In polyglutamine SCAs, ataxia is often associated with extracerebellar signs, including parkinsonism secondary to SNc degeneration [1, 2, 12]. Indeed, neuropathological [1, 13, 14, 15, 16] and positron emission tomography (PET) [13, 14, 15] studies have reported SNc degeneration in these disorders. However, the extent and characteristics of this damage have not yet been well characterized. Questions regarding the possible selective vulnerability of functional territories within the SNc, the onset of nigral degeneration and its progression dynamics, and the correlation between nigral damage and clinical signs remain unclear. As part of the CERMOI multimodal study [4], which included preataxic and early ataxic SCA2 and SCA7 carriers, we aimed to investigate SNc degeneration using neuromelanin‐sensitive imaging. Our objective was to evaluate SNc degeneration as a potential imaging biomarker and to explore its correlation with clinical, imaging, and fluid biomarkers.

## METHODS

### Population

This was a single‐center cross‐sectional prospective study. SCA expansion carriers were recruited consecutively at La Pitié‐Salpêtrière Hospital, Paris, in the setting of the “Integrated functional evaluation of the cerebellum” (CERMOI) study (NCT04288128). The population included SCA carriers who received a genetically confirmed diagnosis of SCA2 (CAG repeat lengths ≥32 in ATXN2) or SCA7 (≥37 in ATXN7), were at least 18 years old, and had a Scale for the Assessment and Rating of Ataxia (SARA) score [17] between 0 and 15/40. Healthy controls needed to test negative for SCA2 and SCA7, have a SARA score below 3, and be free of neurological diseases. The study was approved by the French Ethics Committee and participants provided written informed consent.

### Clinical data

SCA expansion carriers with a SARA <3 were classified as preataxic and those with SARA ≥3 as ataxic [17]. Ataxic participants reported age at ataxia onset (Table 1). Time from ataxia onset was estimated using CAG repeat length for all participants [4, 18]. Additional assessments included the Inventory of Non‐Ataxia Signs (INAS) [19], Cerebellar Cognitive Affective Syndrome Scale (CCAS) [20], and emotional recognition test [21]. Participants were also tested for the presence of parkinsonism signs and upper motor signs. Fasting blood was collected, and plasma neurofilament light chains (NfL) were measured using the Simoa HD‐1 Analyzer (Quanterix) [22].

**TABLE 1:** Clinical and demographical characteristics of the population.

*n*	HC	SCA2			SCA7			Global tests (*p*‐value)	Post hoc tests (*p*‐value)
		Preataxic	Ataxic	All	Preataxic	Ataxic	All		
	10	5	10	15	8	7	15		
Sex (female), *n* (%)	6 (60)	4 (80)	6 (60)	10 (66.7)	2 (25)	5 (71.4)	7 (46.7)	0.61	–
Age (years)	43.2 ± 13.9 [26–65]	38.4 ± 16.9 [21–66]	45.1 ± 8.4 [37–64]	42.9 ± 11.7 [21–66]	41.8 ± 11.9 [26–56]	37.6 ± 15.0 [18–60]	39.8 ± 13.1 [18–60]	0.79	–
CAG repeat length of expanded allele^a^	–	37.2 ± 1.8 [36–40]	38.1 ± 1.3 [35–39]	37.8 ± 1.5 [35–40]	40.0 ± 3.0 [38–47]	48.0 ± 7.6 [39–62]	43.7 ± 6.8 [38–62]	–	–
Disease duration (years)	–	6.0	8.7 ± 6.1 [1–17]	8.4 ± 5.8 [1–17]	6.5 ± 2.1 [5–8]	7.7 ± 4.8 [0–12]	7.4 ± 4.3 [0–12]	0.70	–
Time to onset (years)	–	−7.08 ± 10.2 [−18.1–6]	8.7 ± 5.9 [1–17]	3.4 ± 10.5 [−18–17]	−4.3 ± 13.5 [−29.1–9.9]	7.7 ± 4.8 [0–12]	1.3 ± 11.2 [−29–12]	0.69	–
Upper motor sign, *n* (%)	0 (0)	0 (0)	7 (70)	7 (46.7)	4 (50)	7 (100)	11 (73.3)	**<0.001**	SCA7 > HC**
Parkinsonism, *n* (%)	0 (0)	1 (20)	5 (50)	6 (40)	0 (0)	2 (28.6)	2 (15.5)	0.056	–
SARA (0 = normal, 40 = severe ataxia)	0.1 ± 0.2 [0–0.5]	1.0 ± 1.1 [0–2.5]	7.0 ± 3.1 [3.5–12.5]	5.0 ± 3.9 [0–12.5]	0.7 ± 1.0 [0–2.5]	12.7 ± 5.1 [3.5–19.5]	6.3 ± 7.1 [0–19.5]	**0.001**	SCA2 > HC***; SCA7 > HC**
INAS (0 = normal, 16 = severe non‐ataxic signs)	1.6 ± 1.0 [1–4]	1.8 ± 0.8 [1–3]	4.5 ± 1.7 [2–7]	3.6 ± 2.0 [1–7]	3.0 ± 1.3 [2–5]	5.6 ± 1.6 [3–8]	4.2 ± 1.9 [2–8]	**0.001**	SCA2 > HC*; SCA7 > HC**; Preataxic SCA7 > HC*
CCAS (0 = severe neuropsychological deficit, 120 = normal)	103.5 ± 7.5 [90–114]	100.6 ± 8.6 [92.5–111.5]	87.9 ± 18.2 [45.5–109.5]	92.1 ± 16.6 [45.5–111.5]	106.0 ± 6.01 [95–113.5]	89.64 ± 12.41 [72–100]	98.4 ± 12.5 [72.0–113.5]	0.10	–
Emotional recognition test	29.5 ± 2.9 [22–32]	29.3 ± 1.5 [28–31.5]	27.7 ± 2.4 [23–31.5]	28.2 ± 2.2 [23.0–31.5]	29.3 ± 1.5 [27–31.5]	25.8 ± 3.6 [20.5–31]	27.6 ± 3.2 [20.5–31.5]	0.13	–
Plasma NfL (pg/mL)	5.5 ± 2.2 [2.2–10.2]	12.9 ± 4.0 [9.4–18.4]	14.91 ± 3.49 [9.1–18.9]	14.2 ± 3.7 [9.1–18.9]	13.3 ± 5.5 [6.8–24.2]	27.7 ± 13.2 [16.6–51.6]	20.0 ± 12.0 [6.8–51.6]	**<0.001**	All: SCA2 >HC***, SCA7 >HC***; Preataxic: SCA2 >HC**, SCA7 >HC**; SCA7: ataxic >preataxic**

*Note*: Quantitative variables are summarized as mean ± standard deviation [min‐max] and categorical variables as counts and percentages. Statistically significant effects for global comparisons with Kruskal–Wallis or Fisher's exact tests are shown in bold. Asterisks indicate the significance level of the post hoc comparisons: adjusted *p* < 0.05 (*), adjusted *p* < 0.01 (**), adjusted *p* < 0.001 (***).

Abbreviations: CCAS, Cerebellar Cognitive Affective Syndrome Scale; F, female; INAS, Inventory of Non‐Ataxia Signs; NA, not assigned; M, male; NfL, plasma neurofilaments light chain; SARA, Scale for the Assessment and Rating of Ataxia.

^a^ Pathological CAG repeat threshold: above 32 for the ATXN2/SCA2 allele and 36 for ATXN7/SCA7 allele.

### MRI acquisition

Participants were scanned on a Siemens Prisma 3Tesla MRI system (Siemens Healthineers) using a 32‐channel receive‐only head coil. The MRI protocol included a whole‐brain T_1_‐weighted three‐dimensional acquisition and a T_1_‐weighted two‐dimensional turbo spin‐echo (TSE) acquisition for neuromelanin‐sensitive imaging (Table S1) [8, 9].

### MRI data analysis

#### Neuromelanin‐sensitive images

##### Data processing and segmentation

**FIGURE 1:**
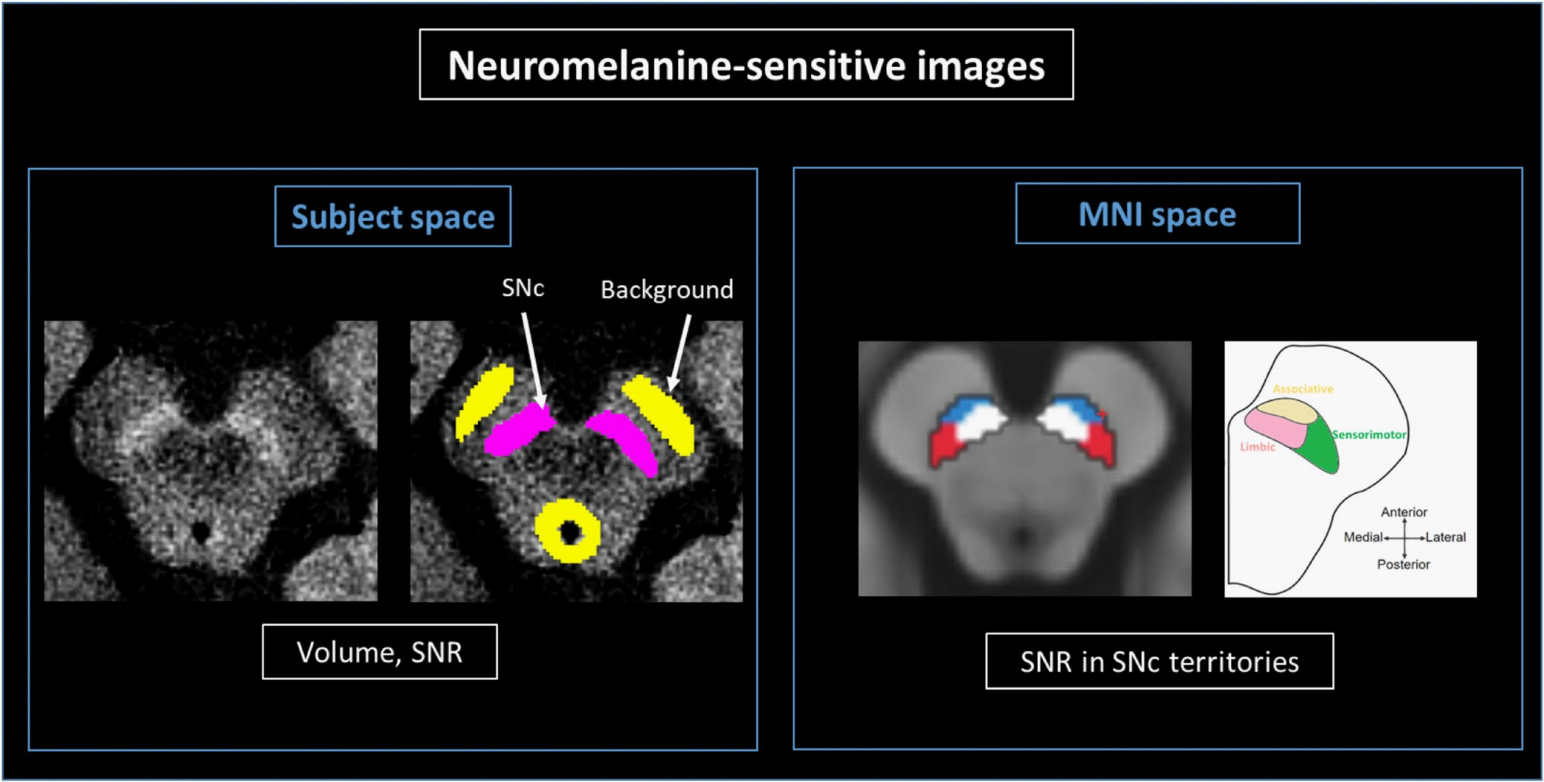
Analysis of neuromelanin‐sensitive images. Left panel: The SNc (purple) and a background region (yellow, including the superior cerebral peduncles and the periaqueductal gray matter) were manually segmented on neuromelanin‐sensitive images to extract the volume and SNR of the SNc. Right panel: Neuromelanin‐sensitive images were registered to a brain template in the MNI space. Then, a mask of SNc with its three functional territories was applied to each neuromelanin‐sensitive image to extract signal values in the SNc territories. Abbreviations: MNI, Montreal Neurological Institute; SNc, substantia nigra *pars compacta*; SNR, signal‐to‐noise ratio.

Similar to previous studies [8, 10, 11], one examiner, blinded to clinical status, manually delineated on neuromelanin‐sensitive images of left and right SN contours as well as a background region covering the superior cerebral peduncles and the periaqueductal gray matter (Figure 1). Intraexaminer reproducibility was assessed using the DICE similarity coefficient.

##### Volume and signal analyses

SNc volumes were calculated by multiplying the voxel size by the number of voxels in the regions of interest (ROIs). T_1_‐weighted images were segmented into white matter, gray matter, and cerebrospinal fluid maps using MATLAB (version R2017b; MathWorks Inc) and Statistical Parametric Mapping (SPM12, v7771) [23] to estimate the total intracranial volume (TIV). SNc volumes were normalized for head size by dividing by TIV. The signal‐to‐noise ratio (SNR) for each slice was calculated by normalizing the mean SNc signal to the background signal using the following formula:

$$\mathrm{SNR}\;=\;\text{mean}\_\text{over}\_\text{slices}\;\left\{\;\left(\text{SigSNc}\;/\;\text{SigBND}\right)\;\right\}\;\times \;100$$

where SigSNc and SigBND are the signal intensities in the SNc and background regions, respectively. For SNc volumes and signal intensity, we used the mean values of the left and right SNc.

##### Analyses in MNI space

This analysis aimed to investigate the spatial pattern of nigral dopaminergic neuron loss. To enable anatomical alignment of SNc segmentations, we used a brain template aligned with the Montreal Neurological Institute (MNI) space [8]. As detailed previously [8, 9, 11], the neuromelanin‐sensitive images were registered to the template for each participant using NiftyReg [24, 25]. Then, we applied to each participant's neuromelanin‐sensitive image previously aligned to the template, an SNc mask manually segmented into three regions based on the functional subdivision of the SNc, including the posterolateral sensorimotor, anteromedial associative, and posteromedial limbic regions, and a background mask [9]. All images were visually inspected for accurate registration and overlay. Signal values from each SNc subregion and the background mask were extracted, and mean SNR values for both SNc sides were calculated for each participant.

#### T_1_ ‐weighted images

Using FreeSurfer 6.0 [26], T_1_‐weighted images were segmented and volumes were derived from 34 bilateral cortical regions using the Desikan‐Killiany atlas and 15 deep brain regions, including the brainstem subregions, basal ganglia, and cerebellum. Volumes were then normalized by TIV.

### Statistical analyses

Statistical analyses were performed using R version 4.3.2 (R Development Core Team, 2023). Continuous data were reported as mean ± standard deviation, and categorical variables as counts and percentages. The level of statistical significance was set at *p* or adjusted *p* < 0.05.

Clinical and demographic data were compared between groups using the Kruskal–Wallis test followed by Dunn's post hoc analysis with a Bonferroni correction for continuous values or the Fisher's exact test for categorical values.

Between‐group differences in SNc volume and SNR were evaluated by fitting multivariate generalized linear models (GLMs, one model per biomarker) using “group” as the main factor, aiming to compare: (i) the three groups, (ii) controls versus preataxic and controls versus ataxic patients, and (iii) ataxic versus preataxic patients. Sex and age were included as covariates as they both influence neuromelanin accumulation in the SNc [27]. Group effects were tested with a type II analysis of variance (*F* test). In case of significant effect, post hoc pairwise comparisons were conducted using Tukey's method. For measurements in MNI space, SNR values in the SNc territories were compared through linear mixed‐effect models (LMMs; one model per biomarker) with age and sex as covariates. Group, region, and their interaction were fixed effects, whereas subject‐specific random intercepts were used to account for repeated measurements of each subject on the ROIs. Significance for the primary and interaction effects of group and region was assessed with type II Wald chi‐square tests. Post hoc pairwise comparisons were performed on a significant interaction or main factor effect, with a correction for multiple comparisons (Tukey's method). For each model, the assumptions and fits were checked.

Receiver operating characteristic (ROC) curves were used to evaluate the performance of biomarkers to discriminate preataxic and ataxic patients from controls in each SCA group. The area under the curve (AUC) of each ROC curve was calculated on the training data set with corresponding 95% confidence intervals (95% CI) using the DeLong's method in the ‘pROC’ package.

Pearson's partial correlations, controlling for age and sex, were performed to investigate relationships between neuromelanin‐derived biomarkers, clinical/NfL variables, and brain volumes. *p*‐values were adjusted for multiple correlations using the false discovery rate (FDR) method.

## RESULTS

### Study population

We enrolled 15 SCA2 patients (ataxic: *n* = 10, preataxic: *n* = 5), 15 SCA7 patients (ataxic: *n* = 7, preataxic: *n* = 8), and 10 age‐ and sex‐matched healthy controls. There were no differences in age, sex, disease duration, or time to onset between the groups. As previously reported [4], SCA2 and SCA7 participants had higher SARA (*p* = 0.001), INAS (*p* = 0.001) scores and plasma NfL levels (*p* < 0.001) than controls. Ataxic SCA7 participants had higher SARA scores (12.7 ± 5.1 vs. 7.0 ± 3.1) and plasma NfL levels (27.7 ± 13.2 vs. 14.91 ± 3.49) than ataxic SCA2 ones, although the difference was not significant. Preataxic patients in both groups also had higher plasma NfL levels than controls (*p* < 0.01).

Parkinsonian signs (including rest tremor and rigidity) were observed in six SCA2 patients (five ataxic and one preataxic) and two SCA7 patients (all ataxic). Upper motor signs (hyperreflexia, spasticity, or extensor plantar reflex) were absent in all preataxic SCA2 patients and were present in four out of eight preataxic SCA7 patients (Table 1).

### Between‐group comparisons of biomarkers

#### Analysis of volumes and signal changes in subject space

The SNc segmentations showed high intra‐rater reproducibility (Dice coefficient: 0.82).

**FIGURE 2:**
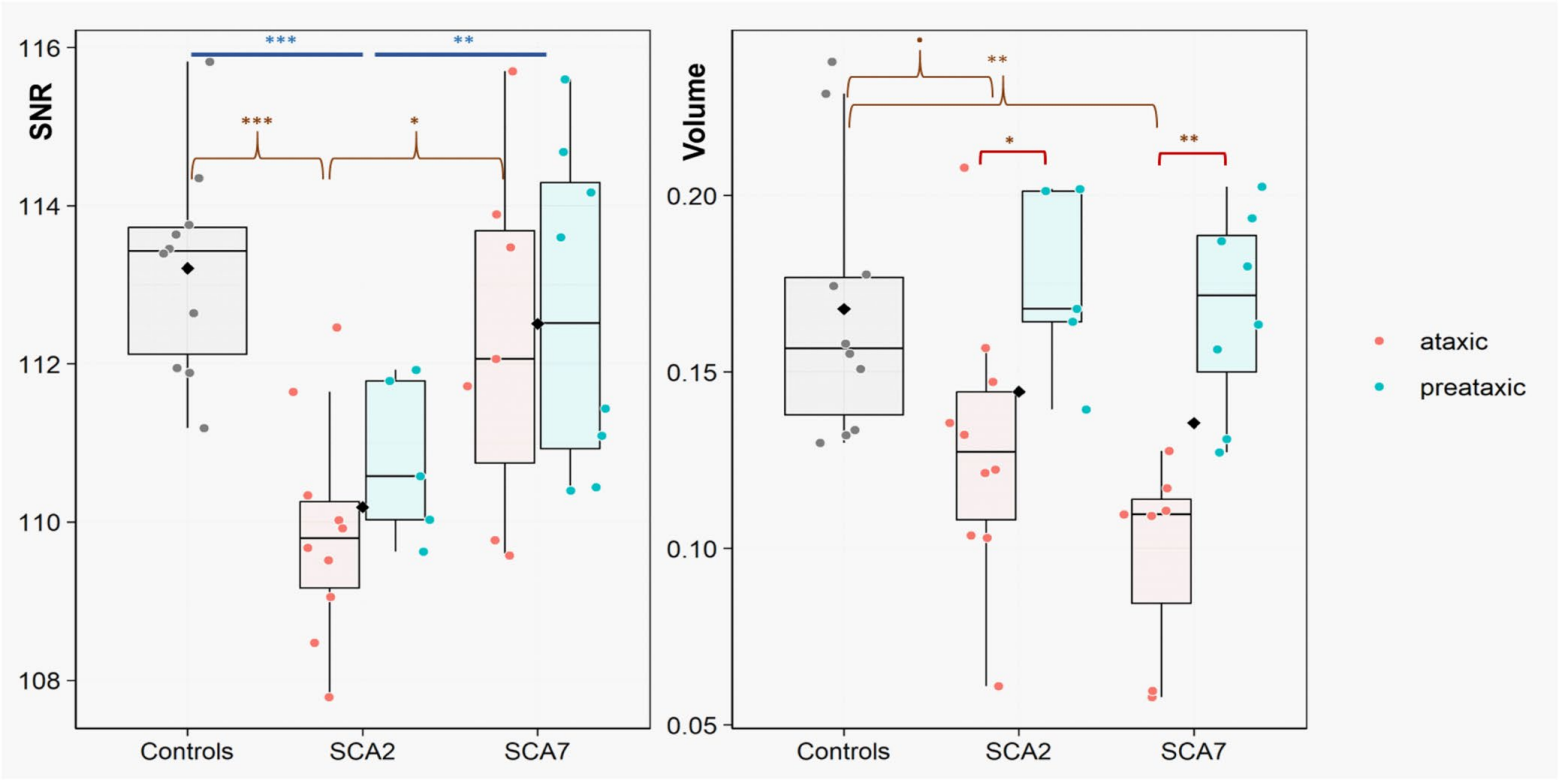
Boxplots representing the distribution of SNR and volume values of the SNc (*y*‐axis) across the different groups (controls, SCA2, and SCA7; *x*‐‐axis) and in ataxic and preataxic subgroups. Each dot corresponds to a subject; black diamonds represent the mean of the group. SNc volume and SNR were compared between groups using multivariate generalized linear models (one model per type of measurement) with sex and age as covariates of no interest, followed by post hoc pairwise comparisons using Tukey's method if a significant difference was found. ***p < 0.001; **0.001 < *p* ≤ 0.01; *0.01 < *p* ≤ 0.05. SNc, substantia nigra pars compacta; SNR, signal‐to‐noise ratio.

Results are summarized in Figure 2 and Table S2.

SCA2 participants (ataxic and preataxic together) had lower SNR values (110.2 ± 1.3) than controls (113.2 ± 1.4; *p* = 0.0003) and SCA7 subjects (112.5 ± 2.1; *p* = 0.003). SNR values did not differ between SCA7 participants and controls. There was a trend for lower SNc volume for SCA2 (0.14 ± 0.04) and SCA7 (0.14 ± 0.04) groups compared to controls (0.17 ± 0.04; GLM: *p* = 0.10).

Ataxic SCA2 (0.13 ± 0.04) and SCA7 (0.10 ± 0.03) subjects had lower SNc volume than controls (0.17 ± 0.04, *p* = 0.06 and *p* = 0.004, respectively). SCA7 patients tended to have lower SNc volume than SCA2 ones (*p* = 0.08). Ataxic SCA2 participants also had lower SNR (109.9 ± 1.4) than controls (113.2 ± 1.4; *p* = 0.0006) and SCA7 subjects (112.3 ± 2.2; *p* = 0.02).

Preataxic SCA2 subjects had intermediate SNR values (110.8 ± 1.0) between ataxic subjects (109.9 ± 1.4) and controls (113.2 ± 1.4), with a trend for lower SNR compared to controls (*p* = 0.07). SNR in SCA7 preataxic subjects (112.7 ± 2.1) did not differ from ataxic ones (112.3 ± 2.2) and controls (113.2 ± 1.4). There was no difference in SNc volume between preataxic subjects and controls.

Ataxic participants had lower SNc volumes than preataxic ones in both groups (SCA2: *p* = 0.02; SCA7: *p* = 0.002). SNR values tended to be lower in ataxic subjects, but the difference was not significant (GLM: *p* = 0.42).

#### Analysis of signal changes by functional territories

**FIGURE 3:**
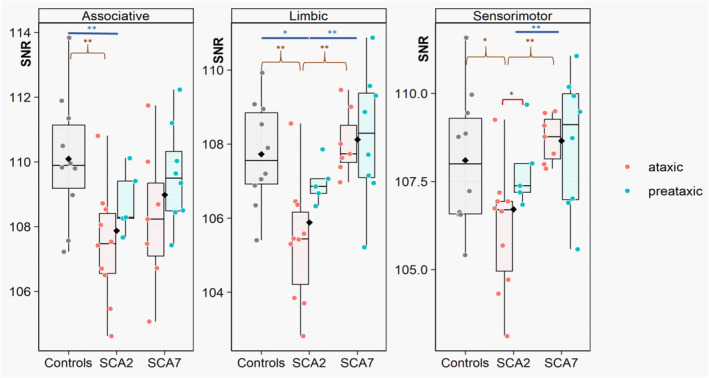
Boxplots representing the distribution of signal values in the territories of the substantia nigra (*y*‐axis) across the different groups (controls, SCA2, and SCA7; *x*‐axis) and in ataxic and preataxic subgroups. Each dot corresponds to a subject; black diamonds represent the mean of the group. SNR values in the sensorimotor, associative, and limbic territories were compared between groups through linear mixed‐effect models (one model per parameter of interest), with age and sex as a covariate of no interest, followed by post hoc pairwise comparisons using Tukey's method if a significant difference was found. **0.001 < *p* ≤ 0.01; *0.01 < *p* ≤ 0.05. SNc, substantia nigra pars compacta; SNR, signal‐to‐noise ratio.

Results are summarized in Figure 3 and Table S2.

SCA2 carriers (ataxic and preataxic together) had lower SNR than controls in the associative (SCA2: 107.9 ± 1.6, controls: 110.1 ± 2.0; *p* = 0.006) and limbic territories (SCA2: 105.9 ± 1.6, SCA7: 107.7 ± 1.4; *p* = 0.02). Values in the sensorimotor territory also tended to be lower compared to controls, but the difference did not reach significance (SCA2: 106.7 ± 1.7, controls: 108.1 ± 1.9, *p* = 0.12). SCA2 carriers had lower SNR than SCA7 in the sensorimotor (SCA2: 106.7 ± 1.7, SCA7: 108.7 ± 1.4; *p* = 0.006) and the limbic territories (SCA2: 105.9 ± 1.6, SCA7: 108.1 ± 1.4; *p* = 0.002). SCA7 subjects did not differ from controls.

In ataxic subjects only, there was a significant difference in SNR in the sensorimotor territory between SCA2 and controls (106.2 ± 1.8 vs. 108.1 ± 1.9, *p* = 0.03).

There was no significant difference between preataxic subjects and controls.

Ataxic patients had lower values than preataxic ones in the sensorimotor territory in SCA2 (106.2 ± 1.8 vs. 107.8 ± 1.1; *p* = 0.049).

#### Relationship to parkinsonism

In SCA2, SNR values were lower in patients with parkinsonism (*n* = 6, 109.5 ± 0.9) compared to those without (*n* = 9; 110.7 ± 1.4), as were the SNc volumes (0.14 ± 0.04 vs. 0.14 ± 0.05, respectively). Similarly, in SCA7, SNR values and SNc volumes tended to be lower in patients with parkinsonism (*n* = 2, SNR: 109.7 ± 0.1, volume: 0.11 ± 0.0) compared to those without (*n* = 13, SNR: 112.9 ± 1.9, volume: 0.14 ± 0.05). No statistical tests were performed due to the low sample size. However, there was a dispersion of values in the group of subjects without parkinsonism with many subjects having low SNR and volume below the mean (Figure S1; Figure 2; Table S3).

#### Brain volumetry

As previously reported [4], compared to controls, SCA2 and SCA7 participants had lower pons (*p* = 0.002 and *p* = 0.02, respectively), medulla oblongata (*p* = 0.003 and *p* = 0.007) and superior cerebellar peduncles (*p* = 0.04 and *p* = 0.004) volumes. SCA2 patients had lower cerebellar volumes than controls (*p* = 0.001) and SCA7 patients (*p* = 0.005). There were no differences in cortical volumes between groups.

Preataxic SCA2 patients had lower pons volume (*p* = 0.01) than controls, and there was a trend for lower medulla oblongata volume (GLM, *p* = 0.06).

Preataxic SCA7 patients did not significantly differ from controls (Figure S3 and Table S4).

### ROC analyses

For the differentiation of SCA2 carriers vs. controls, SNc SNR (ataxic subjects: AUC = 0.96 (95% CI: 0.89–1); preataxic subjects: AUC = 0.94 (95% CI: 0.83–1)) performed as well as plasma NfL (ataxic: AUC = 0.99 (95% CI: 0.96–1); preataxic: AUC = 0.96 (95% CI: 0.87–1)), and pons volume (ataxic: AUC = 0.91 (95% CI: 0.73–1); preataxic: AUC = 0.82 (95% CI: 0.56–1)).

For the differentiation of ataxic SCA7 carriers vs. controls, SNc volume (AUC = 1 (95% CI: 1–1)) had similar performance to plasma NfL (AUC = 1 (95% CI: 1–1)) and pons volume (AUC = 0.94 (95% CI: 0.82–1)). Imaging markers performed poorly in separating preataxic SCA 7 participants from controls (Figure S4 and Table S5).

### Correlation analyses

**FIGURE 4:**
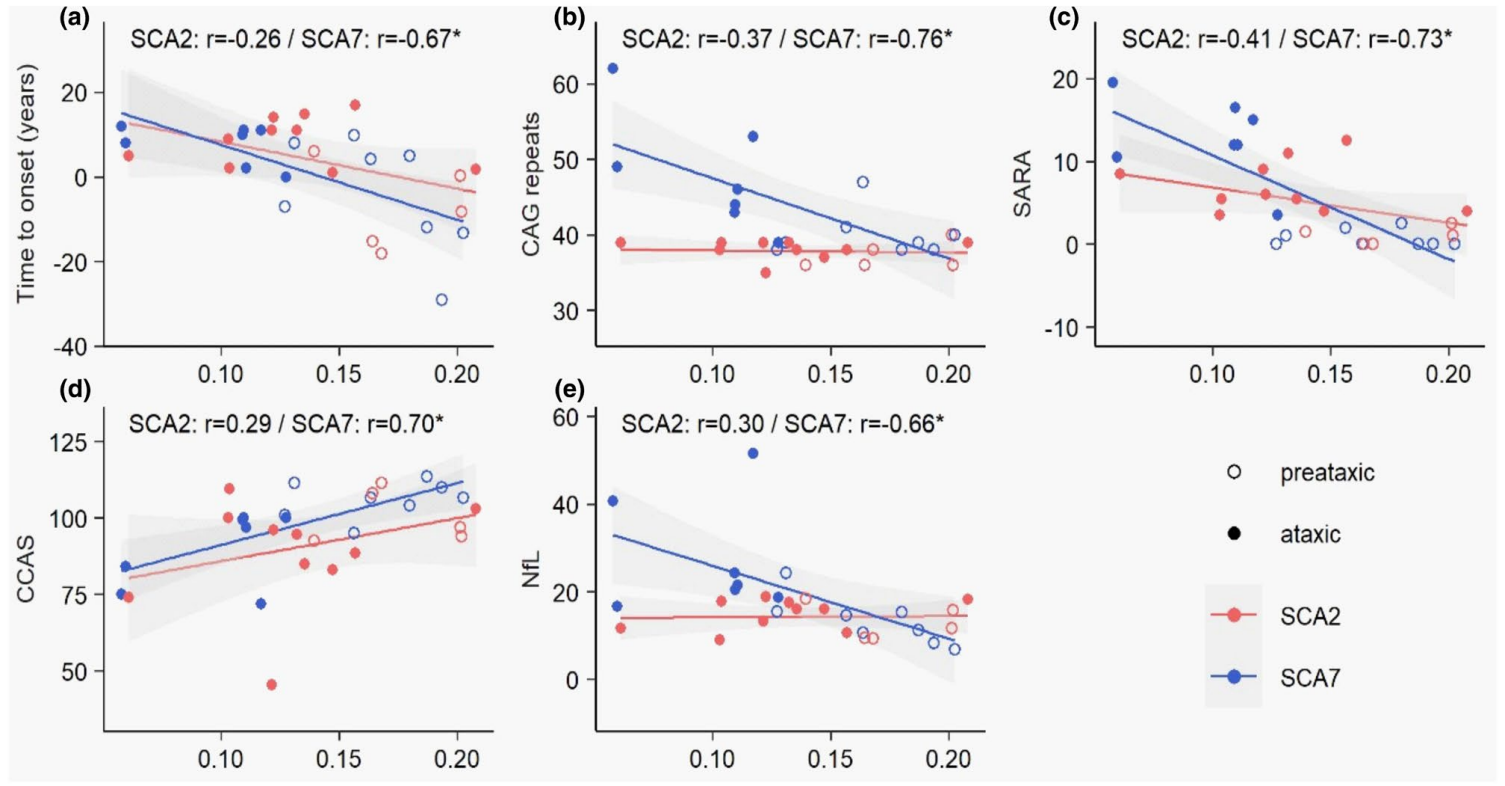
Scatterplots with linear regression lines between SNc volume and correlated clinical variables. Significant correlations between SNc volume and time to onset (a), CAG repeats (b), SARA scores (c), CCAS (d), and plasma NfL (e) were plotted. The shaded grey areas from the “geom_smooth function” of the ggplot2 R package (v3.4.4) represent the 95% confidence area around the fits. The correlations indicated above the plots are Pearson's partial correlations adjusted for age and sex. Correlations with FDR‐adjusted *p*‐values less than 0.05 are marked with an asterisk. Multiple testing was carried out separately for 15 patients with SCA2 (in red) and 15 patients with SCA7 (in blue). Empty and filled points represent respectively patients in the preataxic and ataxic stages. CCAS, Cerebellar Cognitive Affective Syndrome Scale; INAS, Inventory of Non‐Ataxia Signs; NfL: Plasma neurofilaments light chain; SARA, Scale for the Assessment and Rating of Ataxia; SNc, substantia nigra pars compacta; SNR: Signal‐to‐noise ratio.

*In SCA2*, there was a trend before FDR correction toward a correlation between SNR in the associative territory and the emotional recognition test (*r* = 0.63, raw *p* = 0.04, pFDR = 0.60) (Figure 4; Figure S5; Table S6). No significant correlations were observed between neuromelanin‐derived biomarkers and brain volumes.

*In SCA7*, SNc volume was negatively correlated with the estimated time to onset (*r* = −0.67, pFDR = 0.02), pathological CAG repeat expansion (*r* = −0.76, pFDR = 0.01), SARA (*r* = −0.73, pFDR = 0.006), and plasma NfL (−0.66, pFDF = 0.04) and positively correlated with CCAS (0.70, pFDR = 0.02). There was a significant positive correlation between SN volume and nucleus accumbens volume (*r* = 0.79, pFDR = 0.03), as well as a trend for positive correlations between SNc volume and pallidum (*r* = 0.69, pFDR = 0.08), putamen (*r* = 0.69, pFDR = 0.08), and midbrain (*r* = 0.66, pFDR = 0.09) volumes.

There was a trend toward a significant correlation between SNR in the associative territory and the emotional recognition test (*r* = 0.65, raw *p* = 0.003, pFDR = 0.06) and between SNR in the sensorimotor territory and the estimated time to onset (*r* = 0.60, raw *p* = 0.04, pFDR = 0.29) (Figure S6 and Table S7).

## DISCUSSION

Using neuromelanin‐sensitive imaging, we identified nigral alterations in SCA types 2 and 7. SCA2 patients showed reduced SNc neuromelanin signal and volume compared to controls and SCA7 carriers, with greater alterations in ataxic than preataxic subjects. The neuromelanin signal reduction was particularly pronounced in the associative and limbic territories in SCA2, with additional involvement of the sensorimotor territory in ataxic subjects. SCA7 patients had reduced SNc volume, whereas signal values did not differ significantly. The volume decrease was greater in ataxic SCA7 patients compared to both controls and SCA2 patients. Notably, SNc changes were detected at the preataxic stage in SCA2, but not in SCA7. These measurements achieved good discrimination between SCA patients and controls, as did plasma NfL and pons volume. In SCA7, significant correlations were found between neuromelanin‐derived biomarkers and estimated time to onset, pathological CAG repeat expansion, clinical severity scores, and fluid biomarkers.

To date, only two studies used neuromelanin‐sensitive imaging to investigate SNc neurodegeneration in SCAs. One study found that 16 SCA3 patients, including 8 with parkinsonism, had lower SNc neuromelanin signal than controls, with a negative correlation between SNc signal and disease duration [28]. Another study compared nine unspecified SCA patients with parkinsonian groups and controls, finding no significant differences [29]. One case series reported a signal decrease in five patients with SCA31 and parkinsonism [30].

Our findings are consistent with previous PET and neuropathological studies demonstrating nigrostriatal degeneration in various SCA subtypes [13, 14, 15]. Indeed, postmortem studies revealed extensive neuronal loss in the SNc in SCA1 [1, 16, 31], SCA2 [1, 13, 32], SCA3 [1, 13, 16], SCA6 [1, 16, 33], and SCA7 [1, 16, 34, 35]. Similarly, PET studies in SCA2 and SCA3 have reported signs of presynaptic denervation with a reduction in striatal dopamine transporter levels and preservation of postsynaptic striatal D2 receptors, resembling the pattern of idiopathic Parkinson's disease (PD) [13, 14, 15]. From a clinical perspective, a levodopa‐responsive PD‐like phenotype was first described in a patient with SCA3, the most prevalent SCA subtype [36]. Since then, levodopa‐responsive PD‐like and atypical parkinsonism have been reported in many SCA subtypes, including SCA2, SCA6, SCA8, and SCA17 [37]. SCA2 is the subtype most commonly linked to Parkinsonism [37]. In a European series of 163 SCA2 patients, resting tremor was seen in 14.9% of cases, followed by dystonia (14.2%), myoclonus (13.7%), rigidity (7.4%), and chorea/dyskinesia (6.8%) [38]. Parkinsonism in SCA2 being associated with a CAA interruption within the CAG repeat expansion [39], precise sequencing of this expansion could aid in defining the phenotype and improving clinical care. Interestingly, in our study, there was no clear relationship between parkinsonism and SNc alterations, as SNc changes were also observed in participants without parkinsonism. This result was in line with a previous imaging study where no significant difference in neuromelanin signal values was observed between SCA3 patients with and without parkinsonism [28]. Similarly, in another study, eight of 10 patients with SCA3 had significantly decreased ^99^mTc‐TRODAT‐1 uptake. Of these eight, only two had significant parkinsonian signs [15]. In a third study, while severe SNc neurodegeneration was detected in the whole patient sample using dopamine transporter PET and on postmortem analyses, only four out of 19 patients with SCA2 and SCA3 had parkinsonism. Except for one SCA3 patient, patients also had a severe neuronal loss in the subthalamic nucleus, more severe in its motor territory [13]. The authors hypothesized that damage to the motor territory of the subthalamic nucleus could prevent patients with SCA2 and SCA3 from developing parkinsonism despite severe nigral neurodegeneration. This observation is consistent with the fact that selective targeting of the motor territory of the subthalamic nucleus by focal lesions or deep brain stimulation can improve parkinsonian motor features [13].

In our work, SCA2 participants showed greater involvement of the SNc associative and limbic territories. Although SCA participants in our cohort did not exhibit cognitive or emotional disorders on the CCAS and emotional recognition tests, cognitive impairment seen in SCA could be related to dysfunction in the associative or limbic networks. The sensorimotor territory was involved only in ataxic patients. With the involvement of the subthalamic nucleus, this relative preservation of the sensorimotor territory in the preataxic stage may partly explain the absence of parkinsonian signs despite the nigral degeneration. The parkinsonian phenotype may appear later in the disease course when the damage reaches the sensorimotor territory. To our knowledge, no neuropathological study has described the topographical pattern of SNc degeneration. Longitudinal studies at an early stage of the disease, along with the sequencing of the CAG expansion repeat, will enable exploring this hypothesis. This pattern of nigral degeneration is different from that observed in Parkinson's disease and multisystem atrophy, in which the nigral sensorimotor territory projecting to the sensorimotor striatum is involved early, followed by later progression to the associative and then limbic territories as shown in imaging [9, 11] and histological studies [40]. Regarding SCA7 carriers, our study showed a marked reduction in SNc volume in ataxic patients compared to SCA2 ones and controls, without signal decrease. The lower number of ataxic individuals in the SCA7 group compared to the SCA2 one could potentially contribute to the absence of signal reduction detection in SCA7. In postmortem brain studies of SCA patients, somatic instability of the CAG repeat was most pronounced in SCA7 carriers, particularly in the visual cortex and brainstem, including the midbrain where the SN is located. Conversely, SCA2 exhibited the lowest instability index in the midbrain. These findings may also explain the distinct patterns of degeneration observed in SCA7 and SCA2, characterized by volume or signal decreases, respectively. The volume loss appears to correlate with higher levels of somatic instability [41].

In our study, preataxic SCA2 patients had intermediate SNc neuromelanin signal values between controls and ataxic patients, with a trend toward lower SNR compared to controls. This reduction allowed for excellent discrimination of preataxic SCA2 participants from controls. In addition, preataxic SCA2 patients already exhibited pons atrophy compared to controls, with a trend for lower medulla oblongata volume. This result was in line with previous studies in preclinical SCA2 mutation carriers showing marked brainstem atrophy and loss of cerebellar gray matter in lobules V and VI [19, 42]. Conversely, our study did not reveal any significant SNc signal or volume reduction or brain atrophy in preataxic SCA7 carriers.

Using a conservative statistical approach with adjustment for age and sex and correction for multiple comparisons, our study revealed significant correlations in SCA7 between neuromelanin‐derived metrics and critical markers such as estimated time to onset, CAG repeat expansion, clinical severity scores, and plasma NfL. Compared to SCA2, ataxic SCA7 participants had lower SNc volume along with higher SARA scores, plasma NfL levels, and CAG repeat expansion, with a pronounced differential between preataxic and ataxic patients. These results may help explain why significant correlations were observed in SCA7, but not in SCA2.

The main limitation of our study was the small sample size, which resulted from the rarity of SCA2 and SCA7 and the inclusion criterion of a SARA score below 15. This may have reduced statistical power and increased measurement variability. Additionally, the lower number of ataxic SCA7 patients may explain the lack of SNc signal alterations detected in this group. Multicenter studies will allow for larger‐scale investigations, particularly at the preataxic stage, while longitudinal studies will help assess the progression of SNc degeneration over time. Regarding the analysis in template space, aligning neuromelanin‐sensitive images to the template may have caused smoothing in the neuromelanin‐sensitive images, given the different spatial resolution of the two acquisitions, which may have introduced partial volume effects. Some degree of misalignment may have occurred between neuromelanin‐sensitive images and the template, given the small size of SNc relative to the template resolution. However, we implemented measures to mitigate this effect as explained above.

In conclusion, neuromelanin‐sensitive imaging provides evidence of nigral degeneration in SCA2 and SCA7. Specifically, this degeneration was detectable at the preataxic stage in SCA2. In SCA7, neuromelanin‐derived metrics correlated with time to onset, disease severity, and plasma NfL levels. Future research in larger samples and longitudinal datasets will help investigate the progression dynamics of SNc neurodegeneration from the preataxic stage and determine whether such biomarkers could serve as outcome measures in therapeutic trials.

## AUTHOR CONTRIBUTIONS

**Lydia Chougar:** Conceptualization; investigation; writing – original draft; methodology; validation; visualization; writing – review and editing; software; formal analysis; data curation; supervision. **Giulia Coarelli:** Conceptualization; investigation; writing – original draft; funding acquisition; methodology; validation; visualization; writing – review and editing; data curation. **François‐Xavier Lejeune:** Investigation; writing – review and editing; visualization; methodology; validation; formal analysis. **Pia Ziegner:** Investigation; writing – review and editing. **Rahul Gaurav:** Writing – review and editing; formal analysis. **Emma Biondetti:** Formal analysis; writing – review and editing; visualization; validation; methodology; investigation. **Sabrina Sayah:** Writing – review and editing; formal analysis. **Rania Hilab:** Formal analysis; writing – review and editing. **Alain Dagher:** Writing – review and editing. **Alexandra Durr:** Conceptualization; investigation; funding acquisition; writing – review and editing; methodology; project administration; resources; supervision; data curation; formal analysis. **Stéphane Lehéricy:** Funding acquisition; investigation; conceptualization; methodology; validation; visualization; writing – review and editing; project administration; formal analysis; data curation; supervision; resources.

## CONFLICT OF INTEREST STATEMENT

Nothing related to the study. Emma Biondetti has received funding from the European Union's Horizon Europe research and innovation program under the Marie Skłodowska‐Curie grant agreement No 101066055—acronym HERMES. Views and opinions expressed are however those of the author(s) only and do not necessarily reflect those of the European Union or the European Research Executive Agency (REA). Neither the European Union nor the granting authority can be held responsible for them.

## Supporting information


Figure S1.

Figure S2.

Figure S3.

Figure S4.

Figure S5.

Figure S6.

Table S1.

## Data Availability

The data are not publicly available due to privacy or ethical restrictions.
