# Supplementary figures and images for: Substantia nigra degeneration in spinocerebellar ataxia 2 and 7 using neuromelanin‐sensitive imaging
Source: Eur J Neurol. 2025 Jan 5;32(1):e70035. doi: 10.1111/ene.70035 (PMC11702373; doi:10.1111/ene.70035)

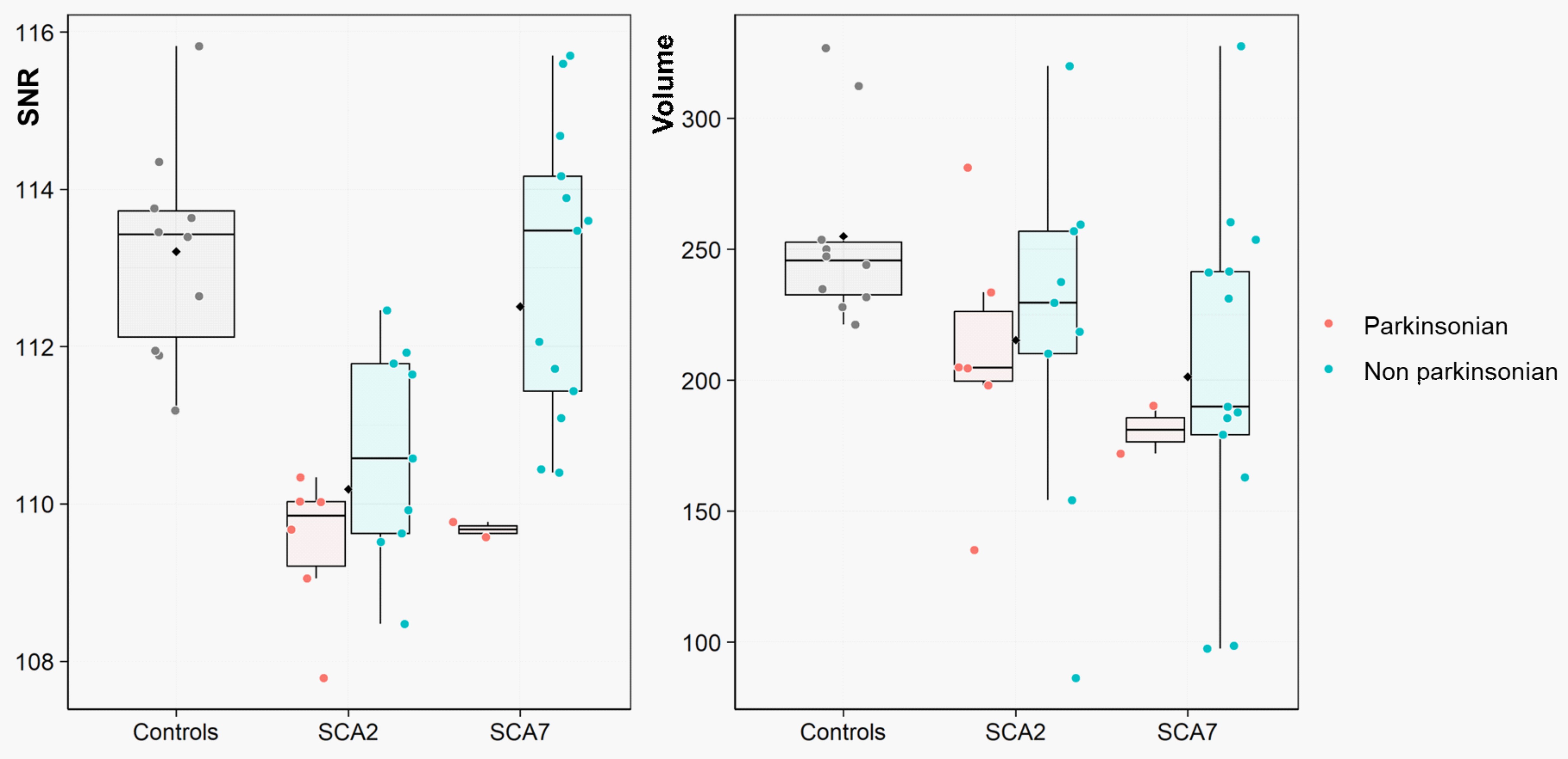

Supplement: Supplementary file 1 — Figure S1. [file ENE-32-e70035-s002.jpg]

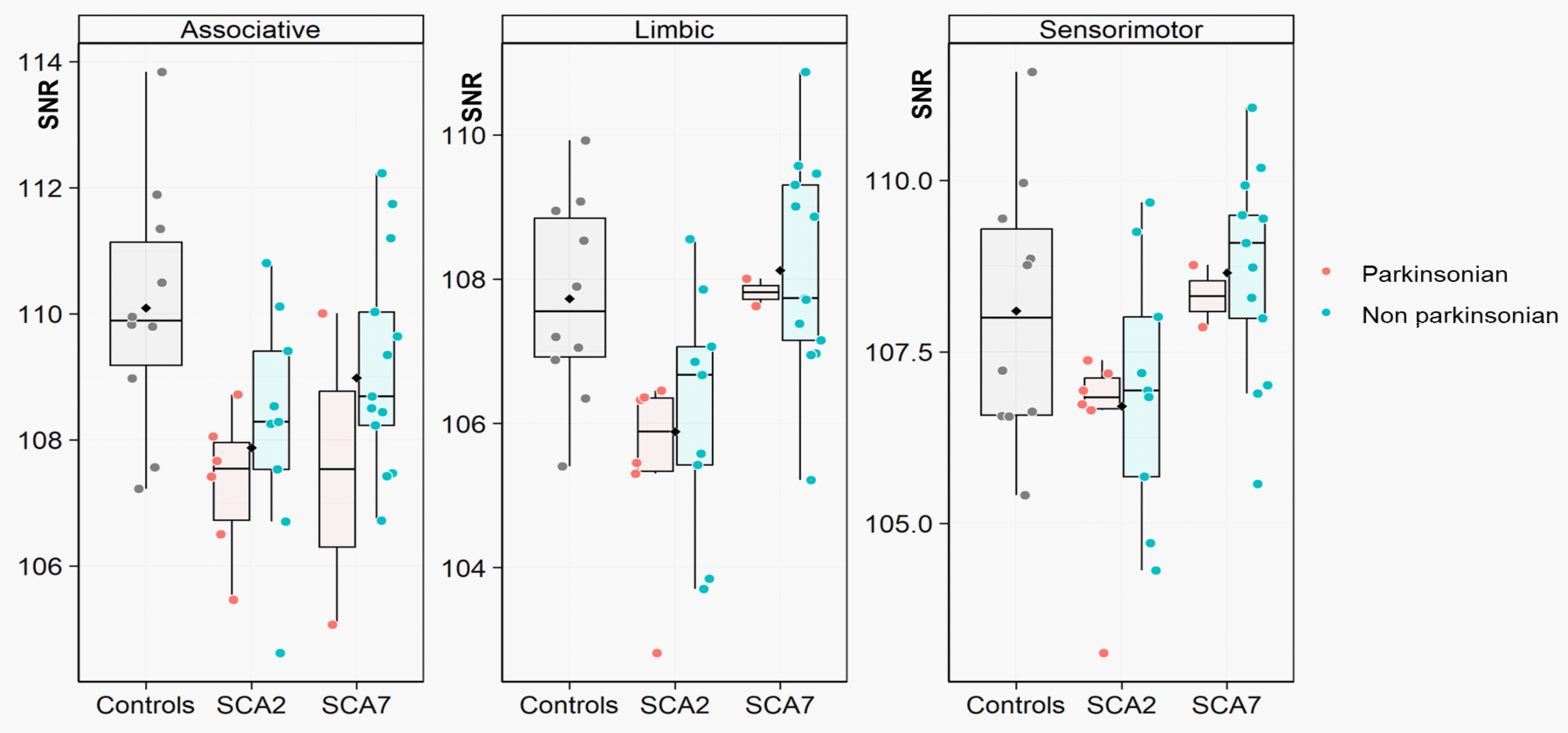

Supplement: Supplementary file 2 — Figure S2. [file ENE-32-e70035-s005.jpg]

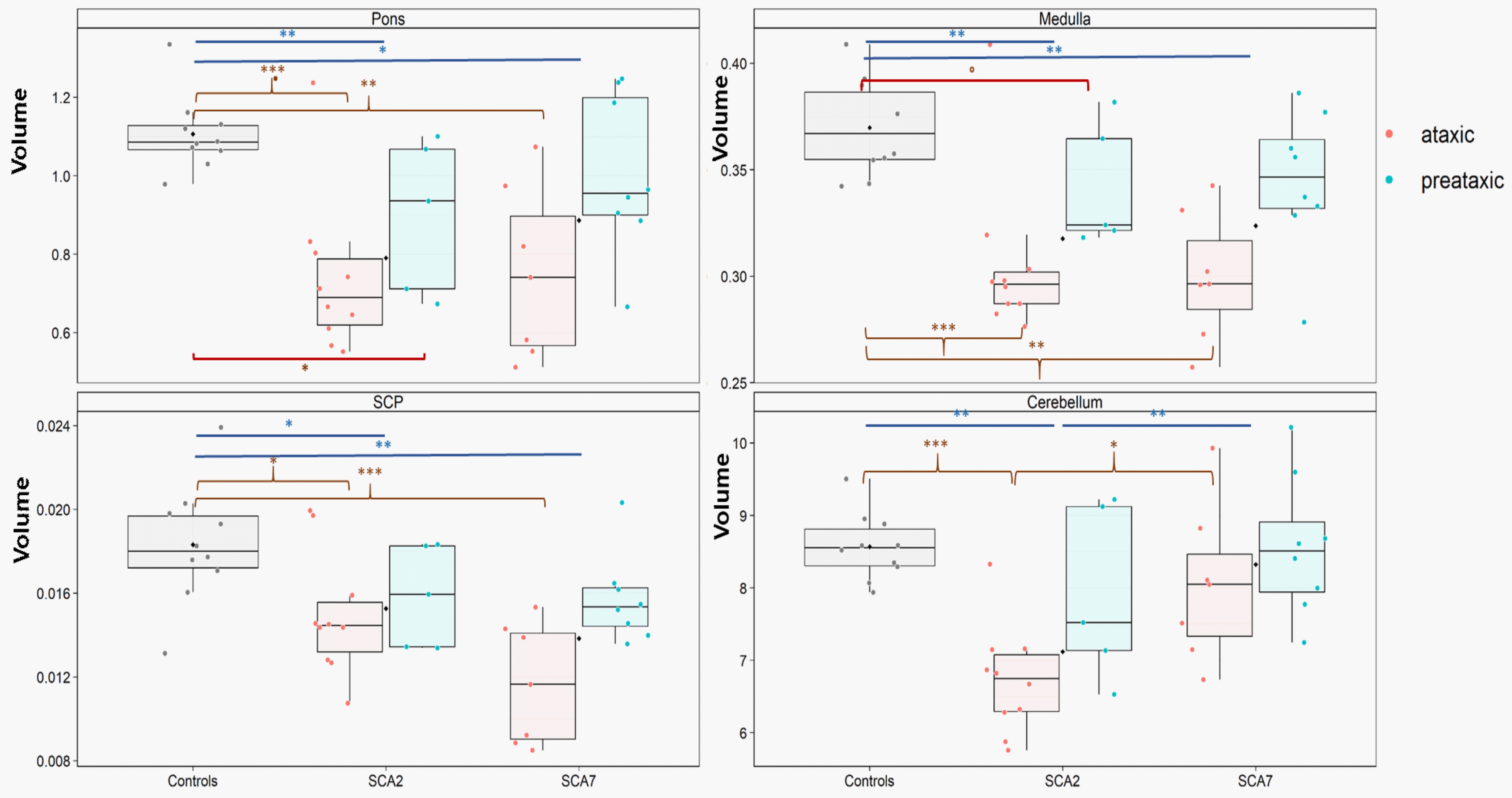

Supplement: Supplementary file 3 — Figure S3. [file ENE-32-e70035-s007.jpg]

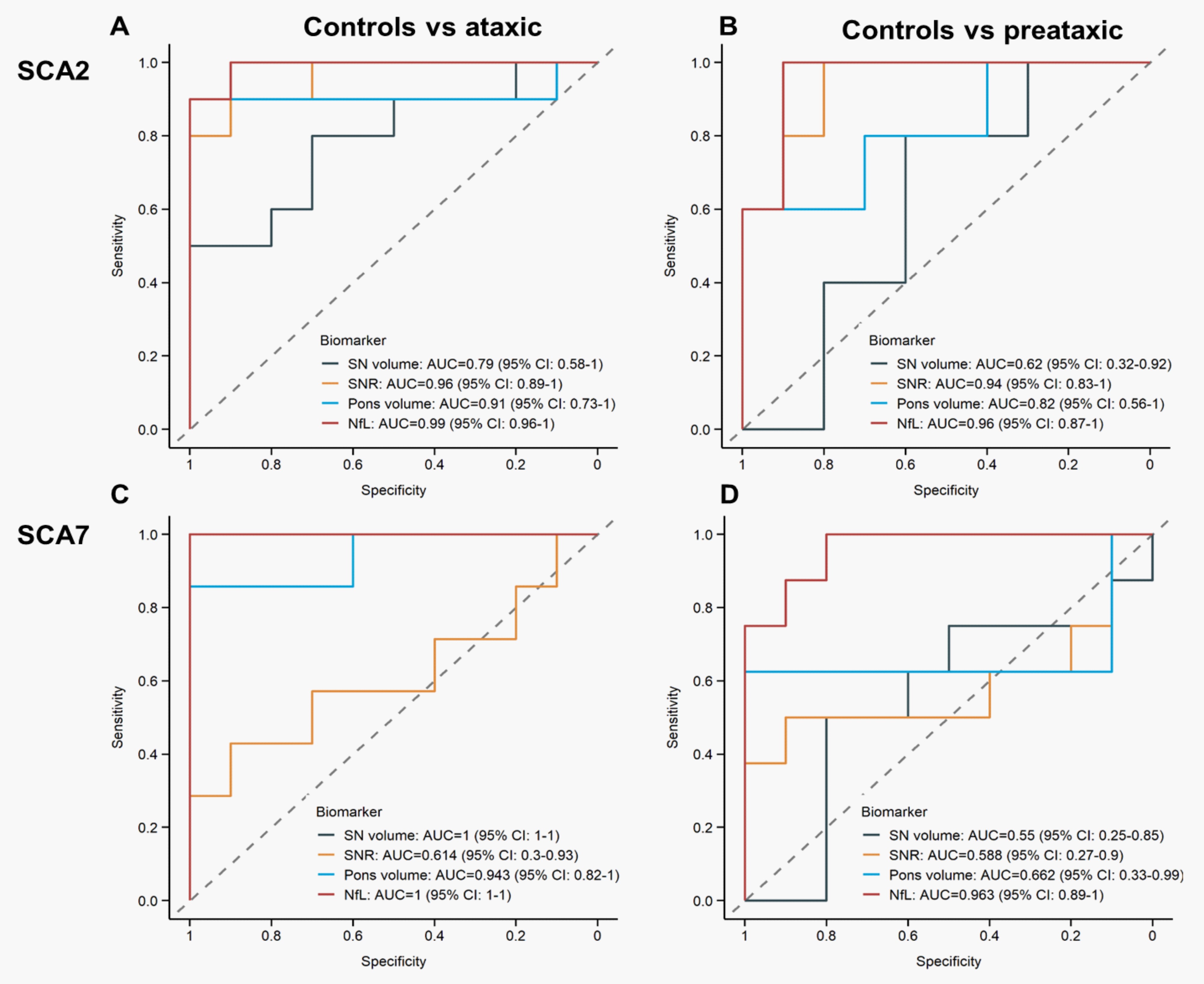

Supplement: Supplementary file 4 — Figure S4. [file ENE-32-e70035-s006.jpg]

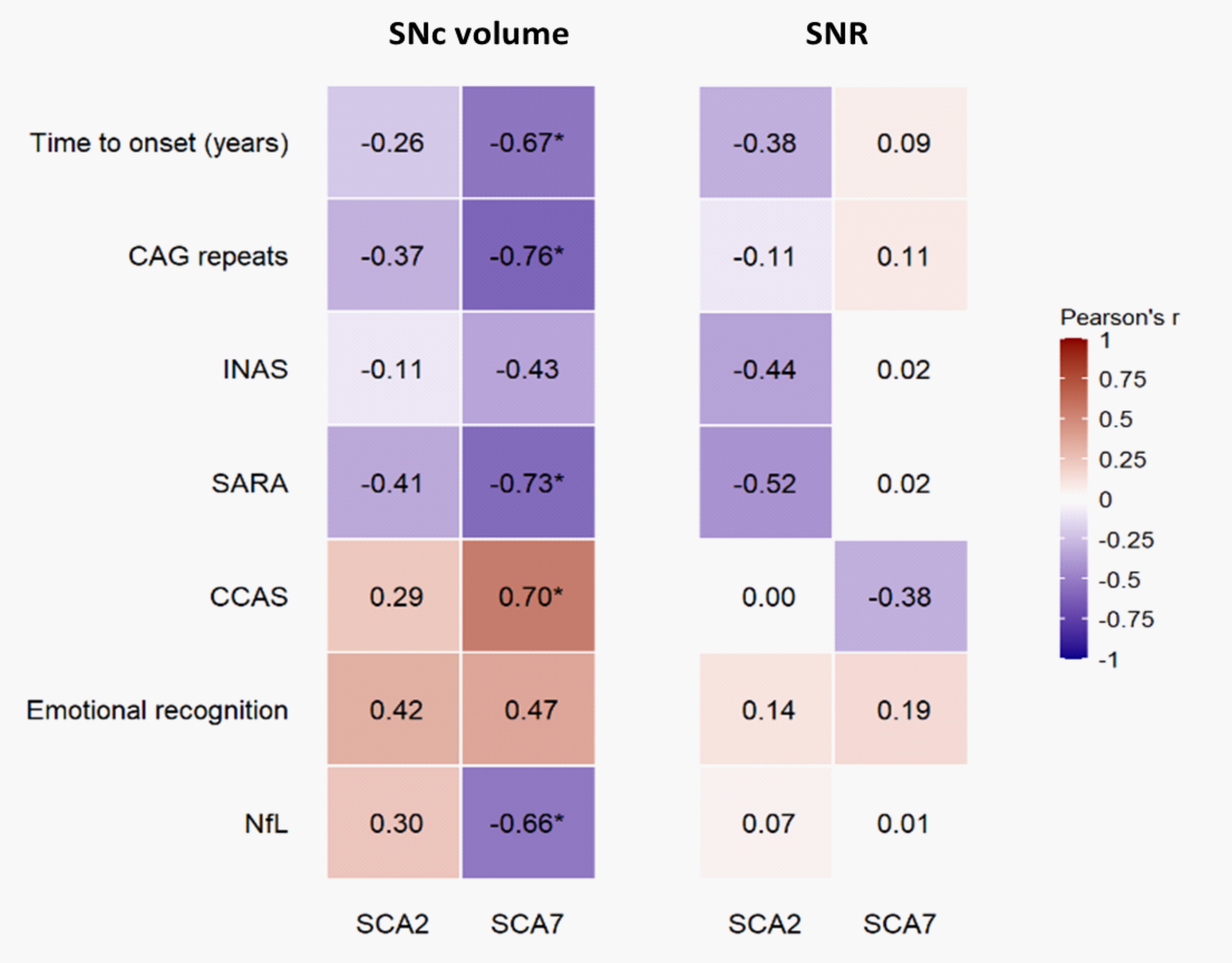

Supplement: Supplementary file 5 — Figure S5. [file ENE-32-e70035-s003.jpg]

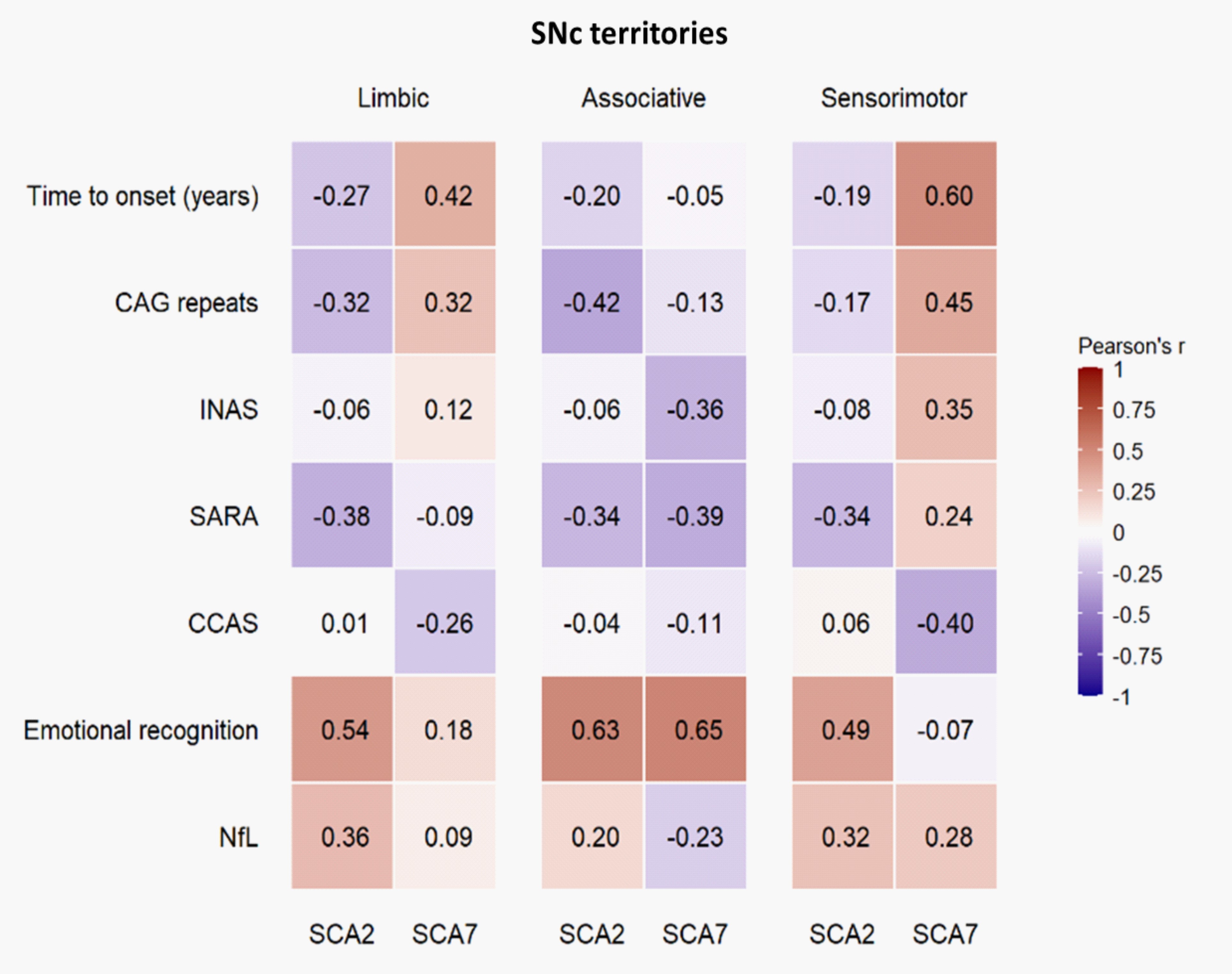

Supplement: Supplementary file 6 — Figure S6. [file ENE-32-e70035-s004.jpg]
