# Supplementary material for: Substantia nigra degeneration in spinocerebellar ataxia 2 and 7 using neuromelanin‐sensitive imaging
Source: Eur J Neurol. 2025 Jan 5;32(1):e70035. doi: 10.1111/ene.70035 (PMC11702373; doi:10.1111/ene.70035)
Supplement: Supplementary file 7 — Table S1. [file ENE-32-e70035-s001.docx]

**Table S1: MRI acquisition protocols**

| **Parameters** | **Sequence:/**  **Units** | **T_1_-weighted** | **T_1_-weighted for neuromelanin-sensitive imaging** |
| --- | --- | --- | --- |
| Pulse sequence type | - | MPRAGE | TSE |
| Imaging type | - | 3D | 2D |
| Echo time (TE) | ms | 2.22 | 13 |
| Repetition time (TR) | ms | 2400 | 890 |
| Inversion times (TIs) | ms | 1000 | - |
| Flip angle | deg | 8 | 90 |
| Echo train length | - | 1 | 3 |
| Pixel bandwidth | Hz/pixel | 220 | 160 |
| Field of view  (RL: right-left; AP: anterior-posterior; FH: foot-head) | mm^3^ | FH x AP x RL: 166 x 240 x 256 | RL x AP x FH: 200 x 220 x 48 |
| Voxel size | mm^3^ | 0.8 x 0.8 x 0.8 | 0.43 x 0.43 x 3 |
| Acquisition matrix size | mm^3^ | 208 x 300 x 320 | 464 x 512 x 16 |
| Slice orientation | - | Sagittal | Transverse |
| Slice angulation | - | - | Perpendicular to the longitudinal axis of the brainstem |
| Phase encoding direction | - | AP | AP |
| Brain coverage | - | Whole brain | Restricted to the midbrain |
| Number of averages | - | 1 | 3 |
| Flow compensation | - | No | Slice-select direction (FH) |
| Acquisition time | min:s | 06:38 | 06:55 |

The table shows the parameters of the T_1_-weighted 3D MPRAGE sequence used for whole-brain anatomical reference and the T_1_-weighted 2D TSE sequence used for neuromelanin-sensitive imaging of the substantia nigra.

**Table S2: Neuromelanin-derived measurements across the different groups and in ataxic and preataxic patients.**

|  | | **Controls** | **SCA2** | | | **SCA7** | | | **Post hoc tests (p value)** |
| --- | --- | --- | --- | --- | --- | --- | --- | --- | --- |
|  |  |  | **Preataxic** | **Ataxic** | **All** | **Preataxic** | **Ataxic** | **All** |  |
|  | **n** | 10 | 5 | 10 | 15 | 8 | 7 | 15 | --- |
| **Whole SNc** | **SNR** | 113.2 ± 1.4  [111.2-115.8] | 110.8 ± 1.0  [109.6-111.9] | 109.9 ± 1.4  [107.8-112.5] | 110.2 ± 1.3  [107.8-112.5] | 112.7 ± 2.1  [110.4-115.6] | 112.3 ± 2.2  [109.6-115.7] | 112.5 ± 2.1  [109.6-115.7] | SCA2<controls***, SCA2<SCA7**; Ataxic SCA2<controls***, Ataxic SCA2<SCA7*; Preataxic SCA2<controls**^.^** |
|  | **Volume** | 0.17 ± 0.04  [0.13-0.24] | 0.17 ± 0.03  [0.14-0.20] | 0.13 ± 0.04  [0.06-0.21] | 0.14 ± 0.04  [0.06-0.21] | 0.17 ± 0.03  [0.13-0.2] | 0.10 ± 0.03  [0.06-0.13] | 0.14 ± 0.04  [0.06-0.20] | SCA2<controls**^.^**, SCA7<controls**^.^**; Ataxic SCA2<controls**^.^**, Ataxic SCA7<controls**; SCA2: ataxic<preataxic*, SCA7: ataxic<preataxic**  Ataxic: SCA7<SCA2**^.^** |
| **SNc territories** | **Associative** | 110.1 ± 2.0  [107.2-113.8] | 108.7 ± 1.0  [107.7-110.1] | 107.4 ± 1.8  [104.6-110.8] | 107.9 ± 1.6  [104.6-110.8] | 109.6 ± 1.6  [107.4-112.2] | 108.3 ± 2.2  [105.1-111.7] | 109.0 ± 1.9  [105.1-112.2] | SCA2<controls**; Ataxic SCA2<controls** |
|  | **Limbic** | 107.7 ± 1.4  [105.4-109.9] | 107.0 ± 0.6  [106.3-107.9] | 105.4 ± 1.6  [102.8-108.6] | 105.9 ± 1.6  [102.8-108.6] | 108.2 ± 1.8  [105.2-110.9] | 108.0 ± 0.9  [107.0-109.5] | 108.1 ± 1.4  [105.2-110.9] | SCA2<controls*, SCA2<SCA7**; Ataxic SCA2<controls**, Ataxic SCA2<SCA7** |
|  | **Sensorimotor** | 108.1 ± 1.9  [105.4-111.6] | 107.8 ± 1.1  [106.8-109.7] | 106.2 ± 1.8  [103.1-109.3] | 106.7 ± 1.7  [103.1-109.7] | 108.6 ± 1.9  [105.6-111.1] | 108.7 ± 0.7  [107.9-109.5] | 108.7 ± 1.4  [105.6-111.1] | SCA2<SCA7**; Ataxic SCA2<controls*, Ataxic SCA2<SCA7**; SCA2: ataxic<preataxic* |

Quantitative variables are summarized as mean±standard deviation [min-max]. Of note, all these measurements are unitless (see Material & Methods). SNc volume and SNR were compared between groups using multivariate generalized linear models (one model per type of measurement) with sex and age as covariates of no interest, followed by post hoc pairwise comparisons using the Tukey’s method if a significant difference was found.

SNR values in the sensorimotor, associative, and limbic territories were compared between groups through linear mixed-effect models (one model per parameter of interest), with age and sex as covariate of no interest, followed by post hoc pairwise comparisons using the Tukey’s method if a significant difference was found. ***p < 0.001; **0.001 < p ≤ 0.01; *0.01 < p ≤ 0.05; **^.^** p<0.10

*Abbreviations: SNc, substantia nigra pars compacta; SNR: signal-to-noise ratio.*

**Table S3: Neuromelanin-derived measurements across the different groups and in parkinsonian and non-parkinsonian patients.**

|  |  | **controls** | **SCA2** | | | **SCA7** | | |
| --- | --- | --- | --- | --- | --- | --- | --- | --- |
|  |  |  | **Preataxic** | **Ataxic** | **All** | **Preataxic** | **Ataxic** | **All** |
|  | **n** | 10 | 5 | 10 | 15 | 8 | 7 | 15 |
| **Whole SNc** | **SNR** | 113.2 ± 1.4  [111.2-115.8] | 110.8 ± 1.0  [109.6-111.9] | 109.9 ± 1.4  [107.8-112.5] | 110.2 ± 1.3 [107.8-112.5] | 112.7 ± 2.1 [110.4-115.6] | 112.3 ± 2.2 [109.6-115.7] | 112.5 ± 2.1 [109.6-115.7] |
|  | **Volume** | 0.17 ± 0.04  [0.13-0.24] | 0.17 ± 0.03  [0.14-0.2] | 0.13 ± 0.04  [0.06-0.21] | 0.14 ± 0.04 [0.06-0.21] | 0.17 ± 0.03 [0.13-0.2] | 0.10 ± 0.03 [0.06-0.13] | 0.14 ± 0.04 [0.06-0.2] |
| **SNc territories** | **Associative** | 110.1 ± 2.0  [107.2-113.8] | 108.7 ± 1.0  [107.7-110.1] | 107.4 ± 1.8  [104.6-110.8] | 107.9 ± 1.6 [104.6-110.8] | 109.6 ± 1.6 [107.4-112.2] | 108.3 ± 2.2 [105.1-111.7] | 109.0 ± 1.9 [105.1-112.2] |
|  | **Limbic** | 107.7 ± 1.4  [105.4-109.9] | 107.0 ± 0.6  [106.3-107.9] | 105.4 ± 1.6  [102.8-108.6] | 105.9 ± 1.6 [102.8-108.6] | 108.2 ± 1.8 [105.2-110.9] | 108.0 ± 0.9 [107.0-109.5] | 108.1 ± 1.4 [105.2-110.9] |
|  | **Sensorimotor** | 108.1 ± 1.9  [105.4-111.6] | 107.8 ± 1.1  [106.8-109.7] | 106.2 ± 1.8  [103.1-109.3] | 106.7 ± 1.7 [103.1-109.7] | 108.6 ± 1.9 [105.6-111.1] | 108.7 ± 0.7 [107.9-109.5] | 108.7 ± 1.4 [105.6-111.1] |

Quantitative variables are summarized as mean±standard deviation [min-max]. Of note, all these measurements are unitless (see Material & Methods). No statistical tests were performed due to the low sample size.

*Abbreviations: SNc, substantia nigra pars compacta; SNR: signal-to-noise ratio.*

**Table S4: Brain volumes across the different groups and in ataxic and preataxic groups.**

|  | **Controls** | **SCA2** | | | **SCA7** | | | **Post hoc tests (p value)** |
| --- | --- | --- | --- | --- | --- | --- | --- | --- |
|  |  | **Preataxic** | **Ataxic** | **All** | **Preataxic** | **Ataxic** | **All** |  |
| **n** | 10 | 5 | 10 | 15 | 8 | 7 | 15 | --- |
| **Midbrain** | 0.44 ± 0.04  [0.38-0.51] | 0.43 ± 0.04  [0.38-0.48] | 0.4 ± 0.04  [0.33-0.48] | 0.41 ± 0.04  [0.33-0.48] | 0.43 ± 0.05  [0.36-0.5] | 0.36 ± 0.04  [0.3-0.42] | 0.4 ± 0.06  [0.3-0.5] | Ataxic SCA2<controls*, Ataxic SCA7<controls** |
| **Pons** | 1.11 ± 0.1  [0.98-1.34] | 0.9 ± 0.2  [0.67-1.1] | 0.74 ± 0.2  [0.55-1.24] | 0.79 ± 0.21  [0.55-1.24] | 1.0 ± 0.2  [0.67-1.25] | 0.75 ± 0.22  [0.51-1.07] | 0.89 ± 0.24  [0.51-1.25] | SCA2<controls**, SCA7<controls*; Ataxic SCA2<controls***, Ataxic SCA7<controls**; Preataxic SCA2<controls* |
| **Medulla** | 0.37 ± 0.02  [0.34-0.41] | 0.34 ± 0.03  [0.32-0.38] | 0.31 ± 0.04  [0.28-0.41] | 0.32 ± 0.04  [0.28-0.41] | 0.34 ± 0.03  [0.28-0.39] | 0.3 ± 0.03  [0.26-0.34] | 0.32 ± 0.04  [0.26-0.39] | SCA2<controls**, SCA7<controls**; Ataxic SCA2<controls***, Ataxic SCA7<controls**; Preataxic SCA2<controls**^.^** |
| **SCP** | 0.02 ± 0.0  [0.01-0.02] | 0.02 ± 0.0  [0.01-0.02] | 0.01 ± 0.0  [0.01-0.02] | 0.02 ± 0.0  [0.01-0.02] | 0.02 ± 0.0  [0.01-0.02] | 0.01 ± 0.0  [0.01-0.02] | 0.01 ± 0.0  [0.01-0.02] | SCA2<controls*, SCA7<controls**; Ataxic SCA2<controls*, Ataxic SCA7<controls**; |
| **Cerebellum** | 8.57 ± 0.46  [7.94-9.51] | 7.91 ± 1.21  [6.53-9.22] | 6.72 ± 0.74  [5.76-8.33] | 7.12 ± 1.05  [5.76-9.22] | 8.56 ± 0.97  [7.24-10.21] | 8.04 ± 1.08  [6.73-9.93] | 8.32 ± 1.02  [6.73-10.21] | SCA2<controls**, SCA2<SCA7**; Ataxic SCA2<controls***, Ataxic SCA2<SCA7* |
| **Vermis** | 0.56 ± 0.05  [0.5-0.62] | 0.56 ± 0.06  [0.5-0.63] | 0.48 ± 0.05  [0.43-0.59] | 0.51 ± 0.07  [0.43-0.63] | 0.56 ± 0.07  [0.47-0.62] | 0.53 ± 0.08  [0.47-0.7] | 0.55 ± 0.07  [0.47-0.7] | Ataxic SCA2<controls* |
| **Accumbens** | 0.05 ± 0.01  [0.04-0.06] | 0.05 ± 0.0  [0.04-0.05] | 0.05 ± 0.01  [0.03-0.06] | 0.05 ± 0.01  [0.03-0.06] | 0.05 ± 0.01  [0.04-0.07] | 0.05 ± 0.0  [0.04-0.05] | 0.05 ± 0.01  [0.04-0.07] | ns |
| **Amygdala** | 0.14 ± 0.01  [0.14-0.16] | 0.15 ± 0.01  [0.12-0.16] | 0.15 ± 0.01  [0.14-0.17] | 0.15 ± 0.01  [0.12-0.17] | 0.15 ± 0.01  [0.12-0.17] | 0.14 ± 0.01  [0.13-0.15] | 0.14 ± 0.01  [0.12-0.17] | Ataxic SCA7<controls* |
| **Basal Forebrain** | 0.06 ± 0.01  [0.05-0.07] | 0.06 ± 0.0  [0.05-0.06] | 0.06 ± 0.0  [0.05-0.07] | 0.06 ± 0.0  [0.05-0.07] | 0.06 ± 0.0  [0.05-0.06] | 0.06 ± 0.0  [0.05-0.06] | 0.06 ± 0.0  [0.05-0.06] | ns |
| **Caudate** | 0.52 ± 0.04  [0.45-0.57] | 0.51 ± 0.05  [0.45-0.57] | 0.49 ± 0.04  [0.42-0.56] | 0.49 ± 0.04  [0.42-0.57] | 0.56 ± 0.08  [0.48-0.69] | 0.52 ± 0.04  [0.47-0.58] | 0.54 ± 0.07  [0.47-0.69] | ns |
| **Hippocampus** | 0.57 ± 0.05  [0.49-0.67] | 0.57 ± 0.04  [0.52-0.62] | 0.6 ± 0.03  [0.55-0.67] | 0.59 ± 0.04  [0.52-0.67] | 0.58 ± 0.03  [0.55-0.63] | 0.56 ± 0.03  [0.51-0.59] | 0.57 ± 0.03  [0.51-0.63] | ns |
| **Pallidum** | 0.2 ± 0.01  [0.19-0.22] | 0.21 ± 0.01  [0.2-0.23] | 0.2 ± 0.02  [0.17-0.23] | 0.21 ± 0.02  [0.17-0.23] | 0.21 ± 0.02  [0.19-0.24] | 0.19 ± 0.01  [0.17-0.21] | 0.2 ± 0.02  [0.17-0.24] | ns |
| **Putamen** | 0.6 ± 0.04  [0.55-0.66] | 0.62 ± 0.04  [0.57-0.68] | 0.59 ± 0.06  [0.52-0.7] | 0.6 ± 0.06  [0.52-0.7] | 0.63 ± 0.04  [0.56-0.69] | 0.56 ± 0.03  [0.5-0.6] | 0.6 ± 0.05  [0.5-0.69] | ns |
| **Thalamus** | 0.86 ± 0.06  [0.78-0.96] | 0.93 ± 0.09  [0.78-1] | 0.87 ± 0.08  [0.77-1] | 0.89 ± 0.08  [0.77-1] | 0.88 ± 0.12  [0.67-1.06] | 0.79 ± 0.06  [0.71-0.86] | 0.84 ± 0.11  [0.67-1.06] | Ataxic SCA7<controls*, Ataxic SCA7<SCA2* |

Quantitative variables are summarized as mean±standard deviation [min-max]. Of note, brain volumes are unitless since they were normalized by the total intracranial volume to TIV to correct for variations in individual head size. Brain volumes were compared between groups using multivariate generalized linear models (one model per type of measurement) with sex and age as covariates of no interest, followed by post hoc pairwise comparisons using the Tukey’s method if a significant difference was found.

***p < 0.001; **0.001 < p ≤ 0.01; *0.01 < p ≤ 0.05; **^.^** p<0.10

**Table S5: Results of the ROC analyses for the classification of controls vs ataxic and preataxic subjects in SCA2 and SCA7.**

|  |  | **Sensitivity** | **Specificity** | **PPV** | **NPV** | **Accuracy** | **Cutoff** | **AUC (CI 95%)** |
| --- | --- | --- | --- | --- | --- | --- | --- | --- |
| **SCA2** | **Ataxic subjects vs controls** | | | | | | | |
|  | **SN volume** | 0.80 | 0.70 | 0.73 | 0.78 | 0.75 | 0.15 | 0.79 (0.58-1) |
|  | **SNR** | 0.90 | 0.90 | 0.90 | 0.90 | 0.90 | 111.8 | 0.96 (0.89-1) |
|  | **Pons volume** | 0.90 | 1 | 1 | 0.91 | 0.95 | 0.91 | 0.91 (0.73-1) |
|  | **NFL** | 1 | 0.90 | 0.91 | 1 | 0.95 | 8.42 | 0.99 (0.96-1) |
|  | **Preataxic subjects vs controls** | | | | | | | |
|  | **SN volume** | 0.80 | 0.60 | 0.50 | 0.86 | 0.67 | 0.16 | 0.62 (0.32-0.92) |
|  | **SNR** | 1 | 0.80 | 0.71 | 1 | 0.87 | 111.9 | 0.94 (0.83-1) |
|  | **Pons volume** | 0.60 | 1 | 1 | 0.83 | 0.87 | 0.96 | 0.82 (0.56-1) |
|  | **NFL** | 1 | 0.90 | 0.83 | 1 | 0.93 | 8.56 | 0.96 (0.87-1) |
| **SCA7** | **Ataxic subjects vs controls** | | | | | | | |
|  | **SN volume** | 1 | 1 | 1 | 1 | 1 | 0.13 | 1 (1-1) |
|  | **SNR** | 0.43 | 0.90 | 0.75 | 0.69 | 0.71 | 111.8 | 0.61 (0.30-0.93) |
|  | **Pons volume** | 0.86 | 1 | 1 | 0.91 | 0.94 | 0.98 | 0.94 (0.82-1) |
|  | **NFL** | 1 | 1 | 1 | 1 | 1 | 13.36 | 1 (1-1) |
|  | **Preataxic subjects vs controls** | | | | | | | |
|  | **SN volume** | 0.50 | 0.80 | 0.67 | 0.67 | 0.67 | 0.18 | 0.55 (0.25-0.85) |
|  | **SNR** | 0.5 | 0.9 | 0.8 | 0.69 | 0.72 | 111.7 | 0.59 (0.27-0.90) |
|  | **Pons volume** | 0.62 | 1 | 1 | 0.77 | 0.83 | 0.97 | 0.66 (0.33-0.99) |
|  | **NFL** | 1 | 0.80 | 0.81 | 1 | 0.89 | 6.73 | 0.96 (0.89-1) |

*Abbreviations: AUC: area under the ROC curve; CI 95%: 95% confidence interval (DeLong’s method); controls: healthy controls; NfL: plasma neurofilaments light chain; NPV: negative predictive value; PPV: positive predictive value; ROC: receiver operating characteristic.*

**Table S6: Correlations between SN volume and SNR and clinical variables, CAG repeats and NfL levels.**

|  | **SCA2** | | | | | **SCA7** | | | | |
| --- | --- | --- | --- | --- | --- | --- | --- | --- | --- | --- |
|  | **lower** | **r** | **upper** | **raw p** | **adj p** | **Lower** | **r** | **upper** | **raw p** | **adj p** |
|  | **Volume** | | | | | | | | | |
| **Estimated time to onset** | -0.712 | -0.265 | 0.335 | 0.110 | 0.307 | -0.893 | -0.673 | -0.193 | 0.006 | **0.021** |
| **CAG repeat length of expanded allele^1^** | -0.764 | -0.368 | 0.23 | 0.790 | 0.945 | -0.923 | -0.758 | -0.354 | 0.004 | **0.018** |
| **SARA** | -0.785 | -0.413 | 0.179 | 0.096 | 0.307 | -0.915 | -0.734 | -0.308 | <0.0001 | **0.006** |
| **INAS** | -0.621 | -0.107 | 0.472 | 0.428 | 0.748 | -0.795 | -0.433 | 0.155 | **0.049** | 0.098 |
| **Emotional recognition** | -0.169 | 0.422 | 0.789 | 0.099 | 0.307 | -0.106 | 0.473 | 0.812 | 0.018 | **0.042** |
| **CCAS** | -0.312 | 0.289 | 0.724 | 0.204 | 0.408 | 0.245 | 0.701 | 0.903 | 0.002 | **0.015** |
| **NfL** | -0.301 | 0.300 | 0.730 | 0.913 | 0.945 | -0.888 | -0.661 | -0.173 | 0.014 | **0.039** |
|  | **SNR** | | | | | | | | | |
| **Estimated time to onset** | -0.769 | -0.379 | 0.218 | 0.147 | 0.343 | -0.484 | 0.092 | 0.612 | 0.942 | 0.942 |
| **CAG repeat length of expanded allele^1^** | -0.624 | -0.111 | 0.468 | 0.635 | 0.945 | -0.470 | 0.109 | 0.623 | 0.587 | 0.806 |
| **SARA** | -0.834 | -0.524 | 0.038 | 0.044 | 0.307 | -0.537 | 0.020 | 0.564 | 0.431 | 0.670 |
| **INAS** | -0.796 | -0.436 | 0.152 | 0.09 | 0.307 | -0.536 | 0.021 | 0.566 | 0.691 | 0.806 |
| **Emotional recognition** | -0.447 | 0.138 | 0.640 | 0.796 | 0.945 | -0.404 | 0.190 | 0.670 | 0.157 | 0.275 |
| **CCAS** | -0.548 | 0.004 | 0.553 | 0.846 | 0.945 | -0.771 | -0.383 | 0.213 | 0.666 | 0.806 |
| **NfL** | -0.498 | 0.074 | 0.600 | 0.945 | 0.945 | -0.541 | 0.015 | 0.561 | 0.904 | 0.942 |

Pearson’s partial correlation coefficients (r) controlling for age and sex are provided with 95% confidence interval, raw and FDR-adjusted p values for each SCA group. Figures in bold indicate significant correlations with raw or FDR-adjusted p values less than 0.05.

*^1^: Pathological CAG repeat threshold: above 32 for the ATXN2/SCA2 allele and 36 for ATXN7/SCA7 allele*

*Abbreviations: NfL: plasma neurofilaments light chain; SARA: Scale for the Assessment and Rating of Ataxia; SN: substantia nigra; SNR: signal-to-noise ratio.*

**Table S7: Correlations between neuromelanin SNR in the SN territories and clinical variables. CAG repeats and NfL levels.**

|  | **SCA2** | | | | | **SCA7** | | | | |
| --- | --- | --- | --- | --- | --- | --- | --- | --- | --- | --- |
|  | **lower** | **r** | **upper** | **raw p** | **adjusted p** | **lower** | **r** | **upper** | **raw p** | **adjusted p** |
|  | **Limbic** | | | | | | | | | |
| **Estimated time to onset** | -0.714 | -0.269 | 0.331 | 0.111 | 0.604 | -0.168 | 0.422 | 0.789 | 0.080 | 0.335 |
| **CAG repeat length of expanded allele^1^** | -0.741 | -0.321 | 0.28 | 0.202 | 0.604 | -0.279 | 0.322 | 0.741 | 0.673 | 0.883 |
| **SARA** | -0.768 | -0.377 | 0.22 | 0.190 | 0.604 | -0.608 | -0.086 | 0.488 | 0.564 | 0.880 |
| **INAS** | -0.593 | -0.063 | 0.506 | 0.567 | 0.758 | -0.465 | 0.115 | 0.626 | 0.552 | 0.880 |
| **Emotional recognition** | -0.016 | 0.54 | 0.841 | 0.321 | 0.653 | -0.412 | 0.18 | 0.665 | 0.325 | 0.759 |
| **CCAS** | -0.547 | 0.006 | 0.555 | 0.700 | 0.842 | -0.711 | -0.262 | 0.338 | 0.999 | 0.999 |
| **NfL** | -0.238 | 0.36 | 0.76 | 0.721 | 0.842 | -0.481 | 0.095 | 0.614 | 0.922 | 0.999 |
|  | **Associative** | | | | | | | | | |
| **Estimated time to onset** | -0.678 | -0.203 | 0.391 | 0.190 | 0.604 | -0.586 | -0.052 | 0.513 | 0.928 | 0.999 |
| **CAG repeat length of expanded allele^1^** | -0.787 | -0.418 | 0.173 | 0.327 | 0.653 | -0.636 | -0.13 | 0.453 | 0.049 | 0.285 |
| **SARA** | -0.748 | -0.336 | 0.264 | 0.183 | 0.604 | -0.772 | -0.386 | 0.21 | 0.054 | 0.285 |
| **INAS** | -0.593 | -0.062 | 0.506 | 0.577 | 0.758 | -0.758 | -0.356 | 0.242 | 0.323 | 0.759 |
| **Emotional recognition** | 0.124 | 0.632 | 0.877 | **0.044** | 0.604 | 0.158 | 0.652 | 0.885 | **0.003** | 0.063 |
| **CCAS** | -0.578 | -0.04 | 0.522 | 0.876 | 0.919 | -0.622 | -0.108 | 0.471 | 0.402 | 0.843 |
| **NfL** | -0.397 | 0.197 | 0.675 | 0.981 | 0.981 | -0.692 | -0.228 | 0.37 | 0.269 | 0.759 |
|  | **Sensorimotor** | | | | | | | | | |
| **Estimated time to onset** | -0.67 | -0.189 | 0.404 | 0.148 | 0.604 | 0.075 | 0.601 | 0.865 | **0.040** | 0.285 |
| **CAG repeat length of expanded allele^1^** | -0.661 | -0.173 | 0.418 | 0.342 | 0.653 | -0.13 | 0.453 | 0.804 | 0.960 | 0.999 |
| **SARA** | -0.752 | -0.344 | 0.255 | 0.230 | 0.604 | -0.361 | 0.237 | 0.697 | 0.973 | 0.999 |
| **INAS** | -0.604 | -0.08 | 0.493 | 0.527 | 0.758 | -0.253 | 0.347 | 0.754 | 0.321 | 0.759 |
| **Emotional recognition** | -0.085 | 0.489 | 0.819 | 0.413 | 0.723 | -0.6 | -0.074 | 0.497 | 0.586 | 0.880 |
| **CCAS** | -0.506 | 0.062 | 0.593 | 0.577 | 0.758 | -0.781 | -0.404 | 0.189 | 0.647 | 0.883 |
| **NfL** | -0.283 | 0.318 | 0.739 | 0.831 | 0.919 | -0.320 | 0.281 | 0.720 | 0.543 | 0.880 |

Pearson’s partial correlation coefficients (r) controlling for age and sex are provided with 95% confidence interval, raw and FDR-adjusted p values for each SCA group. Figures in bold indicate significant correlations with raw or FDR-adjusted p values less than 0.05.

*^1^: Pathological CAG repeat threshold: above 32 for the ATXN2/SCA2 allele and 36 for ATXN7/SCA7 allele*

*Abbreviations: NfL: plasma neurofilaments light chain; SARA: Scale for the Assessment and Rating of Ataxia; SN: substantia nigra; SNR: signal-to-noise ratio.*
